# 15-year experience with rotavirus vaccination in Mexico: a systematic literature review

**DOI:** 10.1080/21645515.2021.1936859

**Published:** 2021-06-30

**Authors:** Adriana Guzman-Holst, Eduardo Ortega-Barria, Ángel Alexis Flores, Roberto Carreño-Manjarrez, Dagna Constenla, Maria Yolanda Cervantes-Apolinar

**Affiliations:** a GSK, Panama City, Panama; b GSK, Mexico City, Mexico

**Keywords:** acute diarrhea, rotavirus gastroenteritis, Mexico, rotavirus vaccine, systematic review, immunogenicity, health economics, disease burden, intussusception

## Abstract

A systematic review was conducted in Mexico to consolidate and evaluate evidence after 15 years of rotavirus vaccination, according to the National Immunization Program. Five databases were screened to identify published articles (January 2000–February 2020) with evidence on all clinical and epidemiological endpoints (e.g. immunogenicity, safety, efficacy, impact/effectiveness) of rotavirus vaccination in Mexico. Twenty-two articles were identified (observational studies including health-economic models: 17; randomized controlled trials: 5). Fourteen studies evaluated a human attenuated vaccine (HRV), four studies evaluated both vaccines, and only two evaluated a bovine-human reassortant vaccine, with local efficacy data only for HRV. Local evidence shows vaccines are safe, immunogenic, efficacious, and provide an acceptable risk-benefit profile. The benefits of both vaccines in alleviating the burden of all-cause diarrhea mortality and morbidity are documented in several local post-licensure studies. Findings signify overall benefits of rotavirus vaccination and support the continued use of rotavirus vaccine in Mexico.

## Introduction

Diarrhea is among the leading causes of mortality in young children under 5 years of age, especially in low- and middle-income countries.^1,2^ Every year, estimated 1.5 million children under 5 years of age die of diarrhea worldwide.^3^ Vaccine-preventable rotavirus infection is one of the most frequent causes of gastroenteritis and diarrhea, which can lead to rapid dehydration in children under 5 years of age.^4,5^ Since 2006, two live, orally administered rotavirus vaccines have been available and licensed for the prevention of rotavirus gastroenteritis: Rotarix® (GlaxoSmithKline Biologicals, Rixensart, Belgium), a two-dose human attenuated vaccine (HRV), and RotaTeq® (Merck & Co. Inc., West Point, PA, USA), a three-dose bovine-human reassortant vaccine (BHRV).^6^ Based on the World Health Organization (WHO) recommendation, rotavirus vaccines have been introduced into the National Immunization Programs (NIP) of several countries, and licensed in more than 100 countries.^6^ Since then, diarrhea-associated mortality has decreased markedly over time, attributable to the widespread use of rotavirus vaccines with other contributing factors such as improvements in diarrhea treatment, sanitation and provision of safe drinking water, and other aspects related to nutrition (breastfeeding practices, vitamin A supplementation).^7^

Yet, rotavirus is still responsible for high levels of diarrhea-related morbidity globally, especially in low- and middle-income nations.^8^ In Latin America and the Caribbean (LAC) region, during the pre-vaccination era, it was estimated that rotavirus caused about 75,000 hospitalizations and 2 million clinic visits per year.^9^ The majority of this burden peaked during the cool winter months.^9,10^ After rotavirus vaccination, in the LAC region, it was observed that in children <5 years of age the number of diarrheal deaths decreased from 32,780 in 2000 to 8,750 in 2013; and deaths due to rotavirus decreased from 11,631 in 2000 to 2,288 in 2013.^9^ In the LAC region, the most common G type of rotavirus is G1, which is responsible for almost half of the rotavirus diarrhea burden, followed by G4, G3, and G9, although regional and temporal variations are significant.^11^ The Pan American Health Organization (PAHO) Technical Advisory Group on vaccine-preventable diseases recommends that countries in this region should continue making efforts to administer rotavirus vaccines as part of their routine vaccination schedules, at the recommended ages according to the vaccine used, usually at 2 and 4 or 2, 4 and 6 months of age. Both of these schedules, particularly the two-dose with HRV which can be completed by 24 weeks of age, foster the early protection for children at the highest risk of severe disease due to rotavirus diarrhea.^10^

Several Latin American countries participated and led the way in pivotal pre-licensure clinical trials. This led to a comprehensive evidence base from a substantial number of rotavirus-specific studies that are available to guide and inform vaccine policy development in the region.^12^ In July 2004, HRV was first registered in Mexico after which it was introduced for routine use into the NIP of several countries in the region.^11^ Brazil and Mexico were among the first to implement childhood rotavirus vaccination into their NIP.^13^ In the Mexican NIP, the two-dose HRV was used from 2006 to 2011 and the three-dose BHRV has been offered since 2011.^14^ The Mexican Social Security Institute (IMSS) partially re-introduced HRV into the NIP in 2019, and distributed both the vaccines through the NIP. It has been over a decade since the licensing and first introduction of rotavirus vaccination into the NIP of Mexico. Numerous studies have been published since, on the impact of vaccination on diarrheal disease burden and safety, testifying to the success of the vaccination programs. Therefore, we conducted a systematic literature review to appraise the available evidence on rotavirus vaccination in Mexico: First we describe findings on the clinical effectiveness, safety, burden of disease, cost-effectiveness of vaccination, and compliance to the recommended vaccine schedule. Then, the results are evaluated to assess the overall impact of rotavirus vaccination on diarrhea-associated mortality, morbidity, and hospitalization since the implementation of the vaccine in the NIP of Mexico (see Figure 6 **Plain Language Summary**).

## Methods

This review was conducted according to the Preferred Reporting Items for Systematic Literature Reviews and Meta-Analyses (PRISMA) guidelines.^15,16^ In line with these guidelines, we developed a search strategy and established study eligibility criteria prior to conducting the review. Following this, searches were performed and retrieved articles were assessed for eligibility in a two-phase screening process and full-text review by two reviewers. From the final list of eligible publications, data were extracted based on the scope which was established *a priori*. A risk of bias assessment was conducted for all included studies independently by two authors.

## Search sources and strategy

The search was conducted in five electronic databases (Medline [via PubMed], EMBASE, Scopus, Latin American and Caribbean Health Sciences Literature [LILACS], and Scientific Electronic Library Online [SciELO]) using a comprehensive set of search terms. The search strategy was developed in Medline, utilizing a combination of both free-text and medical subject headings (MeSH) terms ((Rotavirus) AND (vaccine OR vaccination OR vaccine) AND (Mexico) AND (effectiveness OR impact OR compliance OR safety OR efficacy OR immunogenicity)) and then adapted to the other databases (Supplementary Tables 1). The databases were searched over a 20-year period capturing studies published between January 1, 2000, and February 1, 2020. Articles published in English and Spanish were included in this review and the geographic scope was restricted to Mexico.

## Article selection, data extraction, and reporting

The identified articles were screened in two phases by two reviewers using the inclusion and exclusion criteria provided in Table 1. The retrieved articles were initially screened by title and abstract for eligibility by two reviewers followed by a second step which included screening of the full-text of articles using the eligibility criteria specified in Table 1. Any discrepancies were discussed and resolved with the other review authors.

**Table 1.** Inclusion and exclusion criteria

	Inclusion criteria	Exclusion criteria
Population	Any (not limited to risk groups or specific ages)	Populations with chronic diseases or underlying comorbidities that are not representative of the general population
Intervention	Registered/Licensed rotavirus vaccines in Mexico^±^ • Rotarix® (HRV) • RotaTeq® (BHRV)	All other vaccines
Comparator	All	None
Outcome	Vaccine impact on mortality, morbidity, and hospitalization, burden of disease, immunogenicity, effectiveness, efficacy, safety, cost-effectiveness, compliance	All other outcomes than those specified as eligible
Study design	Primary peer-reviewed research* Observational studies • Cohort studies • Case–control studies • Pre-/post-vaccine introduction time series • Cross-sectional studies • Ecological study Interventional studies • Randomized studies • Non-randomized studies • Cost-effectiveness or health economics studies • Surveys	Non-primary research • Systematic reviews** • Meta-analyses** • Narrative reviews (without methods) Non-human data (e.g. animal models, in-vitro, in-silico) or predictions via modeling methods Case reports Letter to editor Newspaper Editorial Comment Opinions Molecular studies Pilot studies Protocols/pre-clinical studies Studies with insufficient methodological details
Limits		
Publication date	January 1, 2000 to February 1, 2020	All publications outside the eligible time period
Geographic scope	Mexico	All other countries
Language	English, Spanish	All other languages

BHRV, bovine-human reassortant vaccine; HRV, human attenuated vaccine.

^±^For interventional studies.

*References cited by screened articles were manually reviewed for relevance (i.e. snowballing) **References of included articles in these systematic reviews/meta-analyses were manually screened for additional relevant original articles (as deemed necessary by the reviewer).

From each of the eligible articles, relevant information established *a priori* with all authors was extracted using a customized extraction form that included the following items: reference, author, journal and year, region/city, main study objectives, study type/design, study period, sample size, age group, clinical outcomes, and measures of vaccine impact.

A descriptive analysis of the extracted data was performed to summarize the main outcomes of this review.

## Risk of bias assessment

A risk of bias assessment was conducted for all included observational studies and randomized controlled trials (RCTs). The risk of bias for observational studies was assessed using the Strengthening the Reporting of Observational studies in Epidemiology (STROBE)^17^ checklist of essential items, modified according to Sanderson S *et al*. and Fowkes FG & Fulton PM (Supplementary Table 2).^18,19^ An algorithm programmed into a spreadsheet was used to estimate a summary assessment of risk of bias considering five criteria: methods for selecting study participants, methods for measuring exposure and outcome variables, and methods to control confounding, design-specific sources of bias, and statistical methods. The risk of bias of each study was rated as high, moderate, low, or doubtful. The Cochrane risk of bias tool was used to assess RCTs and clinical controlled trials (Supplementary Table 3).^20^ The criteria for judging risk of bias were adequate sequence generation, allocation concealment, blinding, incomplete outcome data, selective reporting, and other sources of bias. The risk of bias of each study was rated as high, moderate and low. The Cochrane Effective Practice and Organization of Care (EPOC) quality criteria were used to assess the risk of bias of the controlled before and after studies and interrupted time series.^21^ The risk of bias assessment was conducted independently by two authors and any disagreements were resolved by consensus through discussion with the authors.

## Results

### Characteristics of included studies

**Figure 1.**
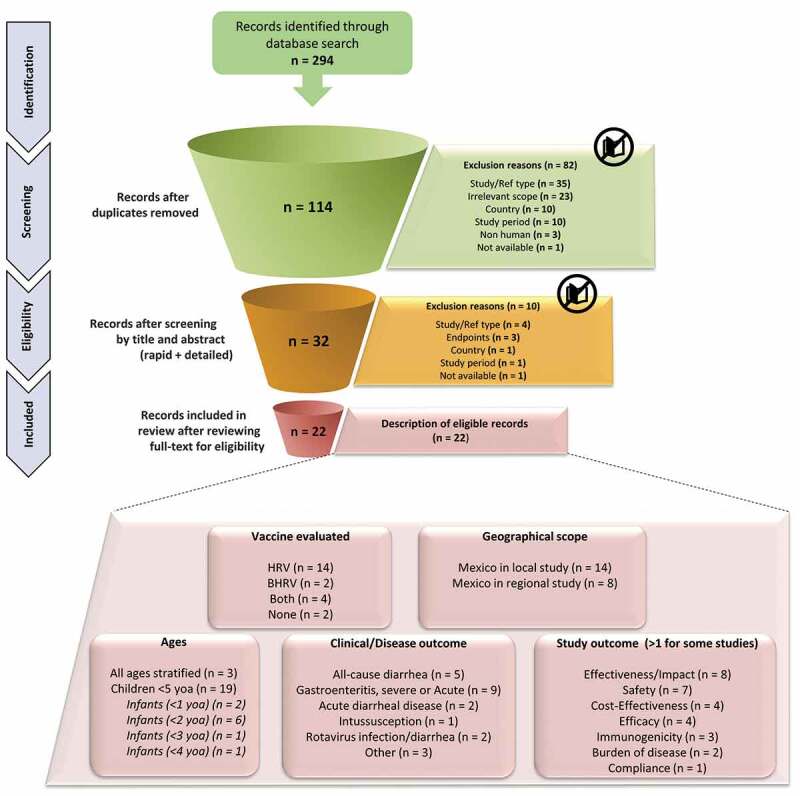
PRISMA flowchart

The literature search yielded 294 articles; of these 114 articles were screened at the title, the abstract phase, and finally 32 articles were screened at the full-text phase (Figure 1). After full-text screening, 22 articles were included in this review (Figure 1; Table 2).^14,22–43^ Among these 22 articles, 14 studies were conducted only in Mexico, whereas eight studies were conducted in the Latin America region and included Mexico (Figure 1; Table 2).

**Table 2.** Overview of main results from the included studies (N = 22)

Reference	Country/state	Time period	Study design	Vaccine	Sample size	Age of study population (years)	Clinical outcome	Main findings
Impact on mortality (n = 5)								
Richardson 2020^37^	Mexico	Pre: 2003–2007 Post: 2007–2017	Observational Study (Database Analysis)	HRV/BHRV	n/a	Children <5 yoa	Acute diarrheal disease	Post universalization period: Mortality decreased by 52.6% During rotavirus seasons: Mortality reduced by 66.9%
Paternina-Caicedo 2015^32^	Mexico (LATAM Study)	Pre: 2002–2005 Post: 2006–2009	Observational Study (Database Analysis)	HRV	n/a	Children <5 yoa	All-cause diarrhea	Relative reduction in death rates: <1 yoa: 35.6 (32.6 to 38.4); <5 yoa: 33.9 (31.4 to 36.3); 702 deaths prevented
Gastañaduy 2012^24^	Mexico	2002–2011; 2003–2006	Observational Study (Database Analysis)	HRV	4,677,341	Children <5 yoa	All-cause diarrhea	Diarrhea Mortality rate reduced: Northern Mexico 45%; Central Mexico 55%; Southern Mexico 43%
Richardson 2010^35^	Mexico	Pre: 2003–2006 Post: 2008–2009	Observational Study (Database Analysis)	HRV	n/a	Children <5 yoa	All-cause diarrhea	December 2007, 74% ≤11 months of age received one dose of rotavirus vaccine. 2008: 1,118 diarrhea-related deaths among <5 yoa; a reduction of 675 from the annual median of 1,793 deaths during the 2003–2006 period. Diarrhea-related mortality, overall: Baseline – 18.1 deaths per 100,000 children 2008: 11.8 per 100,000 children (rate reduction, 35%; 95% CI: 29–39; *P* < .001). Diarrhea-related mortality, ≤11 months of age: Baseline – 61.5 deaths per 100,000 children 2008–36.0 per 100,000 children (rate reduction, 41%; 95% CI: 36–47; *P* < .001). Diarrhea-related mortality between 12 and 23 months: 2008–29% lower for children Mortality among unvaccinated children between the ages of 24 and 59 months was not significantly reduced.
Esparza-Aguilar 2009^26^	Mexico	2000–2007	Observational Study (Database Analysis)	HRV	n/a	Infants (<4 yoa)	Acute diarrheal disease	Deaths in children >5 years of age dropped 42%. Mortality decreased in states that received the vaccine in 2006: 22.7% in >1 yoa 15.8% in 1–4 yoa
Impact on morbidity (n = 3)								
Richardson 2020^37^	Mexico	Pre: 2003–2007 Post: 2007–2017	Observational Study (Database Analysis)	HRV/BHRV	n/a	Children <5 yoa	Acute diarrheal disease	Post universalization period: Hospitalizations decreased by 46% New cases decreased by 15.5% During rotavirus seasons Hospitalizations reduced by 64.7% New cases reduced by 28.7%
Leboreiro 2013^29^	Mexico	2008–2010	Observational Study (Database Analysis)	HRV/BHRV	2,289	Infants (<2 yoa)	All-cause diarrhea	Decrease in the number and severity of rotavirus cases treated in the hospital (OR 0.18, *p* = .01)
Quintanar-Solares 2011^33^	Mexico	Pre: 2003–2006 Post: 2008–2009	Observational Study (Database Analysis)	HRV	Data from 306 censia hospitals	Children <5 yoa	All-cause diarrhea	<5 yoa: Decrease by 11% (N = 9,836) in 2008; decrease by 40% (N = 6,597) in 2009. <1 yoa: Decrease by 25% in 2008, 52% in 2009
Real-world vaccine effectiveness (n = 1)								
Yen 2011^43^	Mexico (Chiapas)	March 2010 – May 2010	Observational (Case-Control)	HRV	14 cases, 29 controls	Children <5 yoa	Severe RVGE	94% effective (95% CI: 16%-100%) after 2nd dose against G9P rotavirus-related hospitalization
Immunogenicity (n = 3)								
Ciarlet 2008^24^	Mexico (LATAM Study)	2005–2006	RCT	BHRV	363	Infants (<2 yoa)	Antibody Responses to OPV and RV	Concomitant administration of BHRV with OPV does not interfere with immunogenicity of each of the poliovirus types 1, 2, and 3.98% of subjects achieved serum-neutralizing antibody titer >1:8 against Poliovirus types (1,2,3) Reduction in the anti-RV IgA titers when given at the same time as OPV, yet children met criteria for seroconversion.
Ruiz-Palacios 2007^38^	Mexico (San Pedro Martir)	June 2001-May 2003	RCT (Phase IIb)	HRV	405	Infants (<2 yoa)	RVGE	Immunogenicity: Seroconversion rates range 34.2–63.9% 2 months after 1st dose and increased to 50–70.6% 2 months after 2nd dose GMC (U/mL) Dose 1 (after 2 months): 36.9 (27.7–49.2); Dose 2 (after 4 months): 58.3 (43.8–77.5); (after 10 months): 102.8 (82.5–128.0)
Salinas 2005^40^	Mexico (LATAM Study)	May 2001 – April 2002	RCT	HRV	2,155	Infants (<1 yoa)	RVGE	Seroconversion rates range 61% (10^4.7^ FFU group) – 65% (10^5,8^ FFU group) 2 months after 2nd dose
Efficacy (n = 4)								
Salinas 2005^40^	Mexico (LATAM Study)	May 2001 – April 2002	RCT	HRV	2,155	Infants (<1 yoa)	RVGE	Against Hospitalization – 79% (95% CI: 48–92%) Severe GE – 86% (95% CI: 63–96%) Any GE – 70% (95% CI: 46–84%) Severe GE (G9 serotypes) – 77% (95% CI: 18–96%)
Ruiz-Palacios 2006^39^	Mexico (LATAM Study)	Pre: 2003–2004	RCT (Phase 3)	HRV	63,225 (safety cohort) 20,169 (efficacy cohort)	Infants (<2 yoa)	Severe RVGE	Severe RVGE and hospitalizations: 85% reduction More Severe RVGE: 100% reduction Hospitalization for diarrhea of any cause: 42% reduction
Ruiz-Palacios 2007^38^	Mexico (San Pedro Martir)	June 2001-May 2003	RCT (Phase IIb)	HRV	405	Infants (<2 yoa)	RVGE	Any GE: 80% (95% CI: 46.3–93.5) Severe GE: 95% (95% CI: 61.0–99.8)
Linhares 2008^31^	Mexico (LATAM Study)	2-year period	RCT	HRV	15,183 (4,335 Mexican)	Infants (<2 yoa)	Severe RVGE	Vaccine Efficacy: Year 1: 82.1% Year 2: 97.3% Year 3: 100%
Safety (n = 7)								
Linhares 2008^31^	Mexico (LATAM Study)	2-year period	RCT	HRV	15,183 (4,335 Mexican)	Infants (<2 yoa)	Severe RVGE	Relative Risk (vaccinated vs placebo) for definite intussusception diagnosed during the first 2 years of life after administration of first vaccine dose was 0.36 (95% CI 0.12–1.06). Intussusception was reported in 4/7,669 (vaccine group) and 11/7,514 (placebo group). No increased risk of definite intussusception was seen in the vaccinated group vs the placebo group during the 2-years’ follow-up (p 0.065)
Salinas 2005^40^	Mexico (LATAM Study)	May 2001 – April 2002	RCT	HRV	2,155	Infants (<1 yoa)	RVGE	The reactogenicity profile of HRV was similar to the placebo, and no vaccination-related serious adverse events were reported. Only 1 intussusception case was reported 6 months after the second dose of HRV (10^4.7^ FFU in a 10-month-old boy)
Ruiz-Palacios 2006^39^	Mexico (LATAM Study)	Pre: 2003–2004	RCT (Phase 3)	HRV	63,225 (safety cohort) 20,169 (efficacy cohort)	Infants (<2 yoa)	Severe RVGE	During the 31-day window after each dose: 6 vaccinated and 7 placebo recipients had definite intussusception (difference in risk: −0.32 per 10,000 infants; 95% CI: −2.91 to 2.18; *P* = .78)
Ruiz-Palacios 2007^38^	Mexico (San Pedro Martir)	June 2001-May 2003	RCT (Phase IIb)	HRV	405	Infants (<2 yoa)	RVGE	SAEs were reported for 31 (8%) of the 405 children, and 2 deaths not related to vaccination
Velázquez 2012^42^	Mexico	January 2008 – October 2010	Observational (Active Surveillance and Controlled Case-Series Analysis)	HRV	221 hospitals (IMSS) 1.5 million infants under surveillance during the study period of almost 3 years	Infants (<1 yoa)	Intussusception	753 episodes of intussusception reported in 750 infants, 701 were in vaccinated infants Post-dose 1: 34.5% Post-dose 2: 65.5% Relative incidence of intussusception within 31 days of vaccination: Post-dose 1: 1.75 (95.5% CI: 1.24–2.48; *P* = .001) Post-dose 2: 1.06 (95.5% CI: 0.75–1.48; *P* = .75) Relative incidence of intussusception within 7 days of vaccination: Post-dose 1: 6.49 (95.5% CI: 4.17–10.09; *P* < .001) Post-dose 2: 1.29 (95.5% CI: 0.80–2.11; *P* = .29)
Baay 2017^22^	Mexico (LATAM Study)	15 February 2016 (Last Accessed) Vaccine Study (Between August 5, 2003, and March 12, 2004)	Observational Study (Database Analysis)	HRV	63,225 (Mexico 13,246)	Infants (<2 yoa)	3 most common SAEs: Gastroenteritis Pneumonia Bronchiolitis	Overall rate of SAEs of 1.05% in all Latin American countries combined (Rate: Min (48.1/10.000 person-years; Max 296.2/10,000 person-years) Overall SAE rates (>1/10,000 person-years) in Mexico: Gastroenteritis – 16.8 Pneumonia – 1.9 Bronchiolitis – 19.5 Bronchopneumonia – 7.5 Bronchitis – 0.2 Bronchospasm – 0.2 Dehydration – 1.9 Urinary Tract Infection – 1.1 Head injury – 1.3 Asthma – 0.2 Febrile Convulsion – 1.5 Pertussis – 0.2 Diarrhea – 0.7 Convulsion – 0.6 Any Preferred Term – 74.3
Ciarlet 2008^24^	Mexico (LATAM Study)	2005–2006	RCT	BHRV	363	Infants (<2 yoa)	Antibody Responses to OPV and RV	Adverse experiences reported within 14 days after any visit: 92.6% in the OPV+RV (concomitant) group vs 89.6% in the RV-OPV (staggered use) group Most commonly reported events (concomitant vs staggered group): Diarrhea (40.2% vs 39.8%) Elevated temperature (24.3% vs 25.8%) Vomiting (21.3% vs 24.9%) Irritability (4.6% vs 6.7%) Vaccine related SAE (concomitant vs staggered group): Intussusception that occurred 3 days after administration of the third dose of RV vaccination in a male infant aged 25 weeks in the staggered use group
Burden of disease (n = 2)								
Linhares 2012^30^	Mexico (LATAM Study)	Mexico: Jan-June 2003	Observational (Active Surveillance Study)	None	Total in Study: 8,031 In Mexico: 1,306 Samples tested: 1,154	Infants (<3 yoa)	Acute and Severe RVGE	For Mexico: Study Duration: 179 days Children enrolled: 1,306 N (samples tested): 1,154 Rota Positive stool samples: 59% (overall)
Granados 2011^28^	Mexico	2001–2006	Observational Study (Database Analysis)	None	n/a	Children <5 yoa	Rotavirus diarrhea	2001: Estimates of DALYS were 19,426 2006: Estimated DALYS decreased by 28.9% Costs of treatment were relatively constant, estimated at US$ 38.7 million and increased only by 5%.
Health economics of rotavirus vaccination (n = 4)								
Carlos 2013^23^	Mexico	5-year period	Health Economic Model	HRV/BHRV	n/a	All ages	Rotavirus infection	Routine immunization with both HRV and BHRV is a highly cost-effective intervention in Mexico. Among the vaccines, HRV was most cost-effective strategy. Without vaccination: 760,559 cumulative rotavirus events in Mexico, entailing direct medical costs of 529.3 million MXN and loss of 5,036 QALYs With HRV: Net Savings of 74 million and gains of 553 QALY with respect to BHRV
Constenla 2009^25^	Mexico	5-year period	Health Economic Model	HRV	The annual birth cohort considered was 2,285,000 children	All Ages	Cost and Health Benefits	Prevent: 651 deaths 13,833 hospitalizations 414,927 outpatient visits Economic Burden: Reduced by US$ 14 million At a vaccine price US$ 16, Cost-Effectiveness Ratio would be US$ 1,139 per DALY
Valencia-Mendoza 2008^41^	Mexico	Projected: 2002–2006 (Unclear)	Health Economic Model	BHRV	Assumption of all birth cohort (2 million)	Children <5 yoa	RVGE	Prevent: 71,464 medical visits (59%) 5,040 hospital admissions (66%) 612 deaths from rotavirus gastroenteritis (70%) Total medical costs prevented: US$74,663.91 (−79,624 to −62,049) At US$10 per dose and a cost of administration of US$13.70 per 3-dose regimen, vaccination would cost: US$122,058 per death prevented US$4,383 per discounted life-year saved Total net cost of US$74.7 million dollars to the healthcare system
Rheingans 2007^34^	Mexico (LATAM Study)	Projected: 2003–2008	Health Economic Model	HRV	n/a	Children <5 yoa	RVGE	Averted with Vaccination vs no Vaccination: Hospitalizations: 651 Deaths: 11,758 Outpatient visits: 412,267 Medical costs: US$11,881,594 (69% reduction) DALYs: 22,330 (70% reduction) ICER (Cost per DALY averted): US$2,070/DALY Meaning: very cost-effective (ICER < US$6,121/DALY), is more than 95%
Compliance (n = 1)								
Luna-Casas 2019^14^	Mexico	2010: HRV 2012: BHRV	Observational Study (Database Analysis)	HRV/BHRV	n/a	All ages	n/a	More eligible infants received all doses with HRV vs BHRV (*p* < .001). Among infants vaccinated with HRV vs BHRV, 93.7% vs 71.1% completed full series (*p* < .001), and 75.5% vs 70.9% completed full series on schedule (*p* = .105), respectively.

BHRV, bovine-human reassortant vaccine; CI, confidence interval; DALY, disability-adjusted life-years; FFU, focus-forming units; GE; gastroenteritis; GMC, geometric mean concentration; HRV, human attenuated vaccine; ICER, incremental cost–effectiveness ratio; IMSS, Mexican Social Security; LATAM, Latin America; n/a, not available; OPV, oral polio vaccine; OR, odds ratio; p, percentage value; QALY, quality-adjusted life-years; RCT, randomized controlled trial; RV, rotavirus vaccination; RVGE, rotavirus gastroenteritis; SAE, serious adverse event; yoa, years of age.

n, sample size; PRISMA, Preferred Reporting Items for Systematic Reviews and Meta-analyses; yoa, years of age.

An overview of the study characteristics is presented in Figure 1 with individual study details provided in Table 2. Of the 22 studies, 17 were observational studies and 5 were RCTs. A majority of the studies included children ≤5 years of age (n = 19 studies) among which the distribution is as follows: infants <2 years (n = 6 studies), <1 year (n = 2), and <3 and <4 years each (n = 1 each).

Eligible studies reported evidence for disease burden (n = 2), immunogenicity (n = 3), efficacy (n = 4), safety (n = 7), impact on all-cause/acute diarrhea mortality and morbidity (n = 7), cost-effectiveness (n = 4), clinical effectiveness (n = 1), and vaccination compliance (n = 1). The majority of studies considered rotavirus gastroenteritis as the clinical endpoint followed by all-cause diarrhea, acute diarrheal disease, rotavirus infection/diarrhea and intussusception.

**Figure 2.**
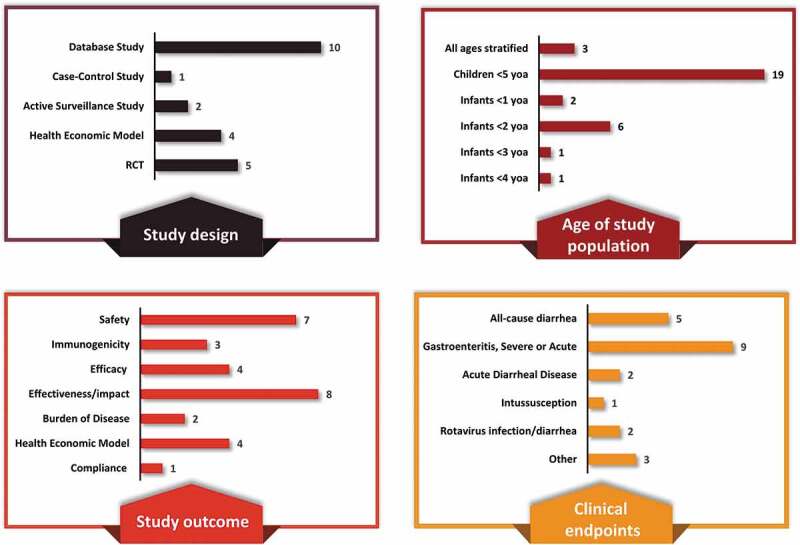
Distribution of studies by (a) Study design, (b) Age of study population, (c) Study outcome, and (d) Clinical endpoint

RTC, randomized control trial; yoa, years of age.

**Figure 3.**
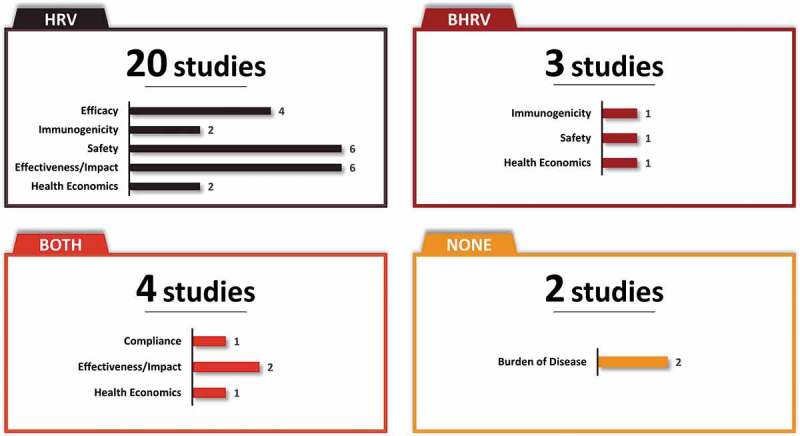
Summary characteristics of studies by vaccine and study outcome*

Fourteen and two studies evaluated HRV and BHRV, respectively, and four studies evaluated both vaccines. The distribution of studies by vaccine and type of study outcome is provided in Figure 2 and Figure 3. Both vaccines, HRV and BHRV, had data for all outcomes with the exception of local efficacy data which were not identified for BHRV.

*Numbers do not add to N = 22 as a study can count toward more than one category (>1 endpoint per study).

### Summary of main findings

#### Burden of disease

One study reported estimates of the burden of disease prior to the implementation of rotavirus vaccination in Mexico in 2006. It estimated the percentage of rotavirus gastroenteritis cases among all-cause acute gastroenteritis cases in children <3 years of age at 59% (2003).^30^ A second study estimated the effect of rotavirus diarrhea on disability-adjusted life-years (DALYS) and diarrhea treatment costs in hypothetical cohorts of infants who were followed from birth up to 5 years of age.^28^ From birth to the age of 5 years, the estimated DALYs were 19,426 in 2001 and decreased by 28.9% in 2006, meanwhile costs of treatment were relatively constant, estimated at US$ 38.7 million and increased only by 5% (Table 2).^28^

#### Immunogenicity and efficacy of rotavirus vaccine

Five RCTs with Mexican participants^24,31,38–40^ provided evidence on the immunogenicity (n = 3)^24,38,40^ and efficacy (n = 4)^31,38–40^ of rotavirus vaccination (Table 2; Figure 3). The immunogenicity of HRV was reported in two studies which showed that most of the infants had seroprotective levels of antibodies when co-administered with the oral polio vaccine and other routine vaccinations.^39,40^ In the first phase 2b, randomized, dose–response study, range of seroconversion rates was 34.2–63.9% 2 months after the first dose and increased to 50%–70.6% two months after second dose in infants 6–12 weeks of age. Geometric mean titers were high and sustained after the completion of two doses.^38^ In a second randomized, placebo-controlled study, the efficacy of different concentrations (10,^4.7^ 10^5.2^ or 10^5.8^ focus-forming units [FFU]) of HRV was evaluated in infants of 6–13 weeks of age. The study reported seroconversion rates of 38% (10^4.7^) to 43% (10^5.8^) two months after the first dose and ranged between 61% (10^4.7^) and 65% (10^5.8^) two months after the second dose.^40^ The immunogenicity of BHRV was reported in one study which lends support to the concomitant use of BHRV and the oral poliovirus vaccine.^24^ While the immunogenicity of OPV did not change when co-administered with BHRV, there was a reduction in the anti-RV IgA titers when given at the same time as OPV, yet children still met the criteria for seroconversion.

Vaccine efficacy was investigated in four studies for HRV.^31,38–40^ Overall, the evidence from RCTs shows that HRV is efficacious in preventing against severe and any rotavirus gastroenteritis, with efficacy ranging from 77%–100% and 70%–80%, respectively.^31,38–40^ The efficacy of rotavirus vaccination against hospitalizations due to any cause of gastroenteritis and severe rotavirus gastroenteritis was 42% and 85%, respectively.^39^ In one study among infants <2 years of age vaccine efficacy was high against severe rotavirus gastroenteritis and sustained up to the third year of life (82.1%–100%).^31^

#### Safety of rotavirus vaccine

A total of seven studies carried out in Mexico provided local evidence of an adequate safety profile of both rotavirus vaccines. While six of the seven studies reported safety data for HRV (randomized [n = 5]; non-randomized[n = 1]),^22,31,38–40,42^ only one provided safety data for BHRV (randomized)^24^ (Table 2). Overall, both vaccines were well tolerated among vaccine recipients with low rates of serious adverse events including a low risk of intussusception. Both vaccines showed an acceptable safety profile when co-administered with the oral polio vaccine and other routine vaccinations.

Significantly fewer serious adverse events were reported among infants who received HRV compared to those who did not receive the vaccine (i.e. placebo).^22,38,40^ While the majority of studies showed that HRV was not associated with an increased risk of intussusception during a 31-day window after administration of the first or second dose versus placebo,^31,38,39^ other studies indicate a low risk of intussusception, specifically a temporal increase in the risk for intussusception within 7 days of administration of the first vaccine dose.^22,42^ In the largest surveillance study for intussusception after rotavirus vaccination to date, the relative incidence of intussusception within 31 days of vaccination was 1.75 (*p* = .001) after the first dose and 1.06 (*p* = .75) after the second dose; and within 7 days of vaccination, the relative incidence was 6.49 (*p* < .001) after the first dose and 1.29 (*p* = .29) after the second dose.^42^ The health benefits of vaccination, in terms of absolute number of deaths and hospitalizations averted, far outweigh the risk of short-term probable side effects which rarely have complications.^44^

A randomized study that evaluated the concomitant use of BHRV with the oral poliovirus vaccine compared to BHRV alone in infants showed a similar safety and tolerability profile between both regimens (Table 2).^24^

#### Health economics of rotavirus vaccination

The cost-effectiveness of rotavirus vaccination in Mexico has been elucidated in four publications (HRV [n = 2]; BHRV [n = 1]; both vaccines [n = 1]).^23,25,34,41^ Overall, the two-dose vaccination schedule with HRV or the three-dose vaccination schedule with BHRV was associated with higher net savings and gain in quality-adjusted life-years (QALY) compared with no vaccination (Table 2).^25,34,41^ Only one analysis directly compared HRV and BHRV.^23^ For both vaccines, the economic evaluation projected a reduction in rotavirus events by 39% for HRV and 30% for BHRV, a reduction in the frequency of cases seeking medical advice by 58% for HRV and 45% for BHRV, and a decrease in hospital admissions by 67% for HRV and 53% for BHRV. The two-dose vaccination schedule with HRV was associated with a net savings of 74 million Mexican pesos (MXN) plus a gain of 553 QALY when compared with the three-dose schedule, with BHRV indicating that vaccination with HRV was the most cost-effective strategy (Table 2).^23^

#### Impact/effectiveness of rotavirus vaccination on mortality and morbidity

Evidence on the impact of rotavirus vaccination on acute diarrheal disease mortality was reported in five studies (HRV [n = 4]; both vaccines [n = 1]) (Table 2).^26,27,32,35,37^ Overall, a substantial decline in all-cause diarrhea mortality rate was observed in children under 5 years of age after the implementation of rotavirus vaccination in Mexico,^26,27,32,35,37^ regardless of the choice of vaccine. The majority of the studies provide evidence of vaccine-specific impact for HRV and one study assessed the overall impact on mortality for a period of 10 years without a differentiation in the vaccine used. During the time when HRV was implemented in the NIP in Mexico, a significant decline in all-cause diarrhea mortality and deaths due to acute diarrheal disease among children under 5 years of age was observed.^26,27,35,37^ Only one study provided evidence of the impact of vaccination with HRV in the different regions of Mexico: across the regions, mortality due to all-cause diarrhea among children aged under 5 years of age declined by 43%–55% in all regions after the implementation of vaccination with HRV (2003–2006) (Table 2).^27^

Evidence on the impact of rotavirus vaccination on all-cause diarrhea morbidity was reported in three studies (HRV [n = 2]; both vaccines [n = 1]) (Table 2).^29,33,37^ Overall, the numbers of new cases and hospitalizations due to all-cause diarrhea including acute diarrhea were reduced during 2006–2017. The first evidence of this comes from a 10-year observational study which showed that rotavirus vaccination resulted in a 15.5%–46% reduction in morbidity (new cases and hospitalizations) resulting from acute diarrheal disease of any cause in children under 5 years old during the post-vaccination period (2008–2017) compared to the pre-vaccination period (2006). This decline was clearly more pronounced (28.7%–64.7%) during the rotavirus season (November–March) in the post-vaccination period.^37^ A study by Leboreiro *et al*. report a reduction in the risk of severe episodes (odds ratio: 0.18, *p* = .01) in children >2 years old, attributable to rotavirus vaccination (both vaccines).^29^ These trends of declining levels of morbidity due to rotavirus vaccination were confirmed in a second study that assessed the vaccine-specific impact of HRV vaccination: a decline of 11%–40% in all-cause diarrhea hospitalizations was observed during 2008–2009 with the greatest reduction reported in infants <12 months of age (25%–52%). In addition, among children 12–23 months of age, a 43% decline in all-cause diarrhea hospitalizations was reported during the 2009 season.^33^

Vaccine effectiveness data were identified only for HRV. In an observational case–control study, a completed 2-dose schedule with HRV resulted in an effectiveness of 94% against hospitalization due to laboratory-confirmed G9P[4] rotavirus infection.^43^

#### Compliance of rotavirus vaccination

Evidence on compliance with the recommended vaccination schedule, including timeliness of vaccination, was reported in one study based on a registry provided by the IMSS. In this registry, there were 659,249 and 780,483 infants eligible for HRV (2010) and BHRV (2012), respectively.^14^ Among these infants, compliance with full vaccine series was reported in 93.7% of infants who received HRV compared to 71.1% who received BHRV (*p* < .001). Likewise, the percentage of infants who completed the full vaccination series according to the recommended schedule (age and interval between doses) was higher with HRV (75.5%) compared to BHRV (70.9%) (*p* = .105).^14^

## Risk of bias

**Figure 4.**
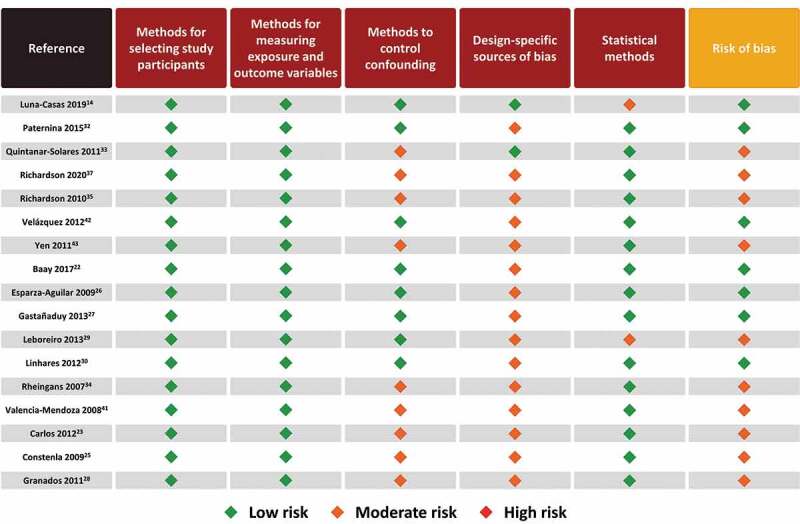
Risk of bias assessment of observational studies using STROBE checklist.^18,19^

The results of the risk of bias appraisal for observational studies are shown in Figure 4. The majority of studies (10/17) were regarded as presenting a moderate risk of bias and the remaining studies presented a low risk of bias. The moderate risk of bias of individual studies was driven mainly by a lack of methods to control confounding and design-specific source of bias which can be attributed to the nature of observational studies, specifically those using passive surveillance and laboratory data (with non-probabilistic sampling methods). Observational studies have inherent biases, particularly since they are not randomized. Yet we classified most as having low-to-moderate risk of biases overall. The specific categories that contained higher bias were mostly around design-specific sources of bias (i.e. recall bias, loss to follow-up, no blinding, retrospective databases from passive surveillance systems, underreporting) and in most studies the methods for controlling confounding (i.e. appropriate design or analytical methods) were unclear/not reported. For almost all of these studies, most endpoints were descriptive with no adjustment for multiple comparisons.

STROBE, Strengthening the Reporting of Observational studies in Epidemiology.

**Figure 5.**
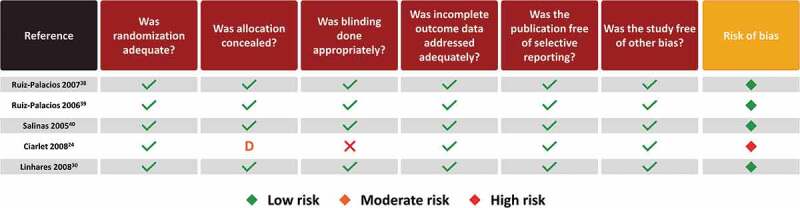
Risk of bias assessment of RCTs using Cochrane risk bias of tool.^20^

The results of the risk of bias appraisal for RCT studies are shown in Figure 5. The majority of studies (4/5) were regarded as presenting a low risk of bias and one study was associated with a high risk of bias.^24^ This was driven by the fact that the concealment of allocation was unclear, and it was not a blinded trial. Adding to this was the small sample size considering loss of follow-up and adherence.^24^

RCT, randomized controlled trial.

**Figure 6.**
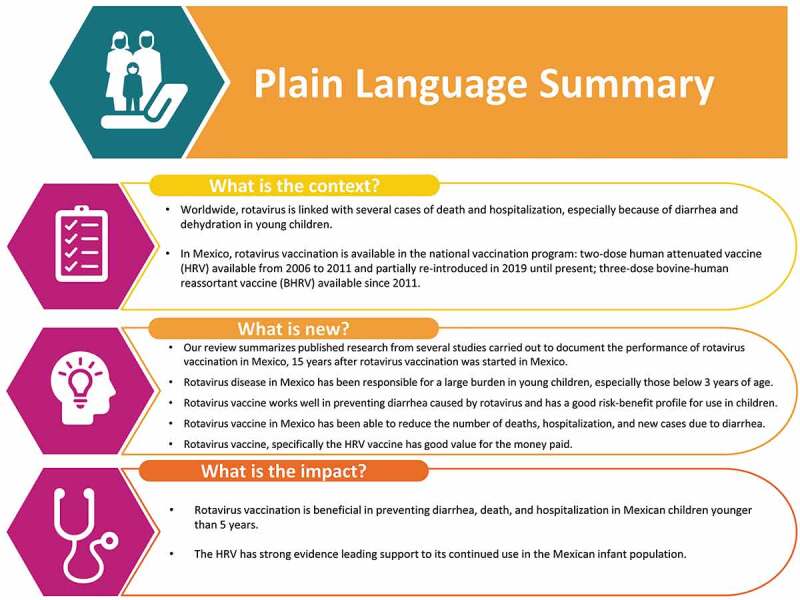
Plain Language Summary

## Discussion

In this review, we summarize evidence on the burden of rotavirus gastroenteritis in Mexico, regional and local immunogenicity, efficacy and safety data of the available rotavirus vaccines, health economics, and the impact of the rotavirus vaccination program in Mexico.

In 2006, rotavirus vaccination for children was added into the Mexican NIP; HRV was used from 2006 to 2011 and partially re-introduced in 2019 until present, and BHRV has been used since 2011. Along with several Latin American countries, Mexico was one of the countries that led the accelerated clinical development of rotavirus vaccines. Since the beginning of the rotavirus vaccination program in Mexico, several studies have been conducted to assess the local immunogenicity, efficacy, and safety of rotavirus vaccines. Local immunogenicity data available only for HRV show that infants had seroprotective levels of antibodies after both vaccine doses,^38,40^ whereas immunogenicity data to support the use of rotavirus vaccines with the oral polio vaccine and other routine vaccinations were available for both HRV and BHRV.^24,38,40^ Local efficacy data were reported in five studies, all of which were specific to HRV.^31,38–40,43^ Overall, the local efficacy of HRV among children <5 years of age is high against severe (77%–100%) and any rotavirus gastroenteritis (70%-80%),^31,38–40^ including hospitalizations (all-cause: 42%; severe rotavirus gastroenteritis-related hospitalizations: 85%).^39^ According to local studies, both rotavirus vaccines show an acceptable safety profile without a severe risk of intussusception. However, a temporal increase in the risk for intussusception was observed within 7 days of receipt of the first vaccine dose.^22,42^ Whether rotavirus vaccination has any impact on the overall incidence of intussusception is yet to be determined.^22,42^ Importantly, this finding should be interpreted along with the well-documented benefits of rotavirus vaccination, demonstrating a high benefit versus risk profile.

Over more than 15 years after implementation of the childhood rotavirus vaccination program in Mexico, a substantial reduction in the diarrheal disease burden primarily among children <5 years of age has been documented. These findings correspond with the trends observed from other Latin American countries such as Brazil and Panama which were also early in their implementation of a national rotavirus vaccination program.^45,46^

In Mexico, G9, a strain fully heterotypic from the vaccine strain, has emerged as an important serotype causing severe rotavirus gastroenteritis.^11,47^ We identified one study that showed high vaccine effectiveness (94%) against laboratory-confirmed G9P[4] rotavirus infection,^43^ indicating that the strain predominance in Mexico was unrelated to vaccine pressure. Because variations in rotavirus types can occur independently of vaccination, the role of vaccination in observed strain changes requires cautious interpretation.^11^

In 2017, a systematic review and meta-analysis was conducted to analyze efficacy, safety, and effectiveness of BHRV and HRV rotavirus vaccines used in the LAC region. This review highlights that the risk of any-severity rotavirus-related gastroenteritis was reduced by 65% following rotavirus vaccination; both vaccines significantly reduced the risk of hospitalization and emergency visits by 85%-90% and did not increase the risk of death, intussusception, or severe adverse events.^48^ Our review reaffirms these previous findings from the region that rotavirus vaccination was effective with a good risk-benefit profile in children. Evidence on compliance to the HRV and BHRV vaccination schedule shows a better compliance (age and interval between doses) with two-dose HRV throughout Mexico, while regional differences were observed with BHRV.^14^

In the majority of health economic evaluations for Mexico, rotavirus vaccination was compared with no vaccination. Only one study that directly assessed the cost-effectiveness of HRV and BHRV was identified in this review; this analysis suggests that vaccination with HRV is a much more cost-effective strategy when compared to vaccination with BHRV.^23^ Findings from health economic evaluations of the rotavirus vaccination program in Mexico underscore the benefit of continuing the rotavirus vaccination program in Mexico. Notably, extensive economic evaluations were performed in the LAC region during the time vaccine introduction decision-making processes were ongoing.^11^ As more data on the vaccine-specific effectiveness of rotavirus vaccination programs become available, further economic analyses are needed to make evidence-based decisions for universal use of rotavirus vaccinations. These analyses would support the ongoing discussions on changing vaccine policy in Mexico based on new epidemiological data or the availability of new rotavirus vaccines.^49,50^

With regard to new rotavirus vaccines, recently, ROTAVAC™ (Bharat Biotech, Hyderabad, India) and RotaSIIL, (Serum Institute of India, Pune, India) received WHO prequalification.^51^ These vaccines are anticipated to expand the global reach of rotavirus vaccines by improving on certain programmatic aspects of HRV and BHRV, like heat stability, reduction of cold-chain footprint, and potentially providing more cost-effective options.^52^ The initial Phase 3 clinical studies of both ROTAVAC and RotaSIIL reported no intussusception events in the first month following any dose of vaccine or placebo; however, these studies are of limited size and geographic scope and thus do not have extensive safety results nor an established risk-benefit profile.^53,54^ These efficacy results are similar to the results from the clinical trials of BHRV and HRV which showed a lower efficacy in low- and middle-income nations with high diarrhea-related mortality. Based on the limited clinical trial data available for these new vaccines, the vaccine efficacy against severe rotavirus disease was 56% for ROTAVAC (in 3 sites in India) and ranged from 37% (in 6 sites in India) to 67% (1 site in Niger) for RotaSIIL.^53–55^ Currently, these vaccines have not been evaluated in Latin American or Mexican populations, and their three-dose schedule might limit their utilization in these countries.

A few limitations of this review are worth noting in the interpretation of the overall findings. Systematic reviews are high in the hierarchy of evidence generation, but they always have specific (inherent) biases such as publication bias. To deal with these biases, we had two reviewers during the screening, and eligibility process and all discrepancies were discussed among the reviewers to reach consensus on the outcome. Additionally, the risk-of-bias evaluations were done as part of the quality assessment of each article in order to reduce biases during the interpretation (i.e. putting less weight on the articles with high risk of bias/lower quality). For this review, we had a wide scope covering a diverse array of clinical and epidemiological endpoints with different time periods considered in the studies. This may have led to the dilution of the individual findings. However, the focus on a single country which has licensed use of both rotavirus vaccines allowed us to meet our review objective to consolidate and integrate all existing evidence on the situation of rotavirus diarrhea in Mexico. Consequently, generalizability to other countries in the region or middle-income countries is limited.

## Conclusions

This systematic review underscores the documented benefit of the childhood rotavirus vaccination program in Mexico more than 15 years after its implementation, specifically in terms of good efficacy/immunogenicity, clinical and real-life effectiveness, a favorable safety and tolerability profile, and substantial reductions in diarrhea-related mortality and hospitalizations. Both HRV and BHRV vaccines have been widely used, and this review highlights that rotavirus vaccines have a large and robust evidence base in Mexico, extending from clinical trials to real-world evidence, and the high compliance rate of HRV with the two-dose schedule, provides confidence in its continued use in all Mexican infants.

## Supplementary Material

Supplemental Material

## Trademark

Rotarix is a trademark owned by or licensed to the GSK group of companies.

RotaTeq is a trademark of Merck Sharp & Dohme Corp.

ROTAVAC is a trademark of Bharat Biotech International Limited.

RotaSIIL is a trademark of the Serum Institute of India.
