# Supplementary material for: 15-year experience with rotavirus vaccination in Mexico: a systematic literature review
Source: Hum Vaccin Immunother. 2021 Jun 30;17(10):3623–37. doi: 10.1080/21645515.2021.1936859 (PMC8437458; doi:10.1080/21645515.2021.1936859)
Supplement: Supplemental Material [file KHVI_A_1936859_SM4353.pdf]

Supplementary material

Supplementary Table 1. General search strategy and database search details

**General Search Strategy (Keywords & String))**			
**Main String**	**Boolean Operator**	**Endpoint String**	**Final Search String**
(Rotavirus) AND (vaccine OR vaccination OR vaccine) AND (Mexico)	**AND**	(effectiveness OR impact OR compliance OR safety OR efficacy OR immunogenicity)	**(Rotavirus) AND (vaccine OR vaccination OR vaccine) AND (Mexico) AND (effectiveness OR impact OR compliance OR safety OR efficacy OR immunogenicity)**

**PubMed** (Interface: National Library of Medicine)
("rotavirus"[MeSH Terms] OR "rotavirus"[All Fields]) AND (("vaccines"[MeSH Terms] OR "vaccines"[All Fields] OR "vaccine"[All Fields]) OR ("vaccination"[MeSH Terms] OR "vaccination"[All Fields]) OR ("vaccin"[Supplementary Concept] OR "vaccin"[All Fields] OR "vaccination"[MeSH Terms] OR "vaccination"[All Fields])) AND ("mexico"[MeSH Terms] OR "mexico"[All Fields]) AND (effectiveness[All Fields] OR ("Impact (Am Coll Physicians)"[Journal] OR "impact"[All Fields]) OR ("safety"[MeSH Terms] OR "safety"[All Fields]) OR efficacy[All Fields] OR immunogenicity[All Fields] OR ("patient compliance"[MeSH Terms] OR ("patient"[All Fields] AND "compliance"[All Fields]) OR "patient compliance"[All Fields] OR "compliance"[All Fields] OR "compliance"[MeSH Terms])))
**EMBASE** (Interface: Elsevier)
('rotavirus'/exp OR rotavirus) AND ('vaccine'/exp OR vaccine OR 'vaccination'/exp OR vaccination OR 'vaccine'/exp OR vaccine) AND ('Mexico'/exp OR Mexico) AND (effectiveness OR 'impact'/exp OR impact OR 'safety'/exp OR safety OR 'efficacy'/exp OR efficacy OR 'immunogenicity'/exp OR immunogenicity OR compliance)
**LILACS** (Interface: BVS)
(tw:(rotavirus)) AND (tw:((vaccine OR vaccination OR vaccine))) AND (tw:((effectiveness OR impact OR safety OR efficacy OR immunogenicity))) AND (tw:(Mexico))
**SciELO Mexico**
rotavirus [Todos los indices] and mexico [Todos los indices]
**Scopus** (Interface: Elsevier)
TITLE-ABS-KEY ((rotavirus) AND (vaccine OR vaccination OR vaccin) AND (mexico) AND (effectiveness OR impact OR safety OR efficacy OR immunogenicity))

Supplementary Table 2. STROBE tool for assessing susceptibility to bias of observational studies[17-19]

Criteria category	Domain	Tool item must address	Risk of Bias# (H, M, L, D)
**Major***	*Methods for selecting study participants	Appropriate source population (cases, controls and cohorts) and inclusion or exclusion criteria	
	*Methods for measuring exposure and outcome variables	Appropriate measurement methods for both exposure(s) and/or outcome(s)	
	*Methods to control confounding	Appropriate design and/or analytical methods	
**Minor**	Design-specific sources of bias (excluding confounding)	Appropriate methods outlined to deal with any design-specific issues such as recall bias, interviewer bias, biased loss to follow or blinding	
	Statistical methods (excluding control of confounding)	Appropriate use of statistics for primary analysis of effect	

*Around half of the checklists included what we regard as the three most fundamental domains of appropriate selection of participants, appropriate measurement of variables and appropriate control of cofounding.

STROBE, Strengthening the Reporting of Observational studies in Epidemiology

#Risk of Bias

H (High risk of bias) clearly indicates bias

M (moderate risk of bias) suggests potential bias

L (low risk of bias) clearly excludes bias

D (Doubtful risk of bias) suggests doubts about potential bias

Summary judgment of the study: High, Moderate, or Low risk of bias

- **High risk of bias:** ≥1 of any criteria clearly (H) indicates bias, or ≥2 major criteria suggest potential bias (M) or doubts (D)
- **Moderate risk of bias:** ≥2 of any criteria suggests potential bias (M) or doubts (D) (<2 major criteria)
- **Low risk of bias:** Low (L) risk of bias in all major criteria and <2 of minor criteria suggests potential bias (M) or doubts (D)

Supplementary Table 3. Cochrane risk of bias assessment tool for RCTs[20, 21]

Author, year [ref]	Was randomization adequate?	Was allocation concealed?	Was blinding done appropriately?	Was incomplete outcome data addressed adequately?	Was the publication free of selective reporting?	Was the study free of other bias?	Risk of bias
**Criteria for judging risk of bias**<br>- Was the allocation sequence adequately generated? [Short form: Adequate sequence generation?]<br>- Was allocation adequately concealed? [Short form: Allocation concealment?]<br>- Was knowledge of the allocated interventions adequately prevented during the study? [Short form: Blinding?]<br>- Were incomplete outcome data adequately addressed? [Short form: Incomplete outcome data addressed?]<br>- Are reports of the study free of suggestion of selective outcome reporting? [Short form: Free of selective reporting?]<br>- Was the study apparently free of other problems that could put it at a risk of bias? [Short form: Free of other bias?]							

Risk of Bias	Interpretation	Relationship to individual criteria
**Low Risk of Bias**	Plausible bias unlikely to seriously alter the results	All of the criteria YES
**Moderate Risk of Bias**	Plausible bias that raises some doubt about the result	One of the criteria UNCLEAR
**High Risk of Bias**	Plausible bias that seriously weakens confidence in the results	One or more criteria NO

RCT, randomized controlled trial
